# Recombinant *Arthrobacter* β-1, 3-glucanase as a potential effector molecule for paratransgenic control of Chagas disease

**DOI:** 10.1186/1756-3305-6-65

**Published:** 2013-03-14

**Authors:** Christo Jose, Nicole Klein, Sarah Wyss, Annabeth Fieck, Ivy Hurwitz, Ravi Durvasula

**Affiliations:** 1 Center for Global Health, Department of Internal Medicine, University of New Mexico and New Mexico VA Health Care System, Albuquerque, New Mexico, USA; 2 Present address: Medical Scientist Training Program, Carver College of Medicine, The University of Iowa, 2206 MERF, Iowa City, Iowa, 52242-2600, USA

## Abstract

**Background:**

Chagas disease is most often transmitted to humans by *Trypanosoma cruzi* infected triatomine bugs, and remains a significant cause of morbidity and mortality in Central and South America. Control of Chagas disease has relied mainly on vector eradication. However, development of insect resistance has prompted us to develop a paratransgenic strategy to control vectorial transmission of *T*. *cruzi*. Here, the potential role of recombinant endoglucanases as anti-trypanosomal agents for paratransgenic application is examined. The surface of *T*. *cruzi* is covered by a thick coat of mucin-like glycoproteins that have been proposed to play a role in the binding of *T*. *cruzi* to the membrane surface of the vector gut. We hypothesize that disruption of these glycoconjugates could arrest parasite development in the vector and abort the transmission cycle. In this work, we examine the effects of recombinant *Arthrobacter luteus* β-1, 3-glucanase expressed via *Rhodococcus rhodnii* on *T*. *cruzi* Sylvio II strain.

**Methods and results:**

The coding sequence for β-1, 3-glucanase was cloned in-frame to a heterologous promoter/signal sequence from the *Mycobacterium kansasii* alpha antigen gene resident in an *E*. *coli*/*R*. *rhodnii* shuttle vector. The resulting construct was confirmed by sequencing, and electroporated into *R*. *rhodnii*. Expression products from positive clones were purified from log phase cultures followed by dialysis into physiological buffers. Lysates and media were quantitated by ELISA against rabbit antibody specific to β-1,3-glucanase. Glucanase-positive samples were applied to live *T*. *cruzi* parasites in culture and viability accessed by spectrophotometric and fluorescent microscopic measurements. *R*. *rhodnii*-expressed β-1,3-glucanase exhibited toxicity against *T*. *cruzi* compared to controls when applied at 5 and 10% of the total culture volume. The decrease in cell viability ranged from a maximum of 50% for the media treatments to 80% for the filtered lysates.

**Conclusions:**

These results suggest that recombinant β-glucanase could be a powerful addition to the arsenal of effector molecules for paratransgenic control of Chagas disease. In future studies, the ability of β-glucanase to function in combination with other effector molecules will be explored. Dual targeting of *T*. *cruzi* should not only slow resistance but also permit synergistic or additive lethal effects on *T*. *cruzi*.

## Background

Chagas disease, a parasitic disease endemic to South and Central America, is caused by the protozoan *Trypanosoma cruzi* and transmitted to humans via the feces of triatomine bugs. It affects approximately 8–11 million people, results in 50,000 deaths and accounts for the loss of 500,000 disability-adjusted life-years annually [1]. Cases of Chagas disease have expanded globally, and are now reported in the Americas, Europe, Australia and Asia due to emigration of infected individuals from endemic parts of the world [2].

Chagas disease manifests in three distinct phases - acute, indeterminate and chronic. Infected individuals present in an initial acute phase that develops into a chronic phase in 20-40% of the patients. The chronic phase is characterized by the development of lesions in the nervous tissue of the heart, intestine and esophagus that result in progressive organ enlargement with potentially fatal complications. Patients who do not progress to the chronic phase of the disease exist in an indeterminate phase [3]. The two drugs used worldwide in the treatment of Chagas disease, benznidazole and nifurtimox, are effective only against the acute stage of the disease [4]. These treatments are marred by their many side effects including bone marrow toxicity, neural toxicity and severe nausea and vomiting [5]. Moreover, strains of *T*. *cruzi* that are resistant to both drugs have emerged and resistance to one drug is usually coupled with resistance to the other [6].

Several efforts have been undertaken to control transmission of the disease to human populations from triatomine vectors. These transmission-prevention programs include insecticide-based campaigns, housing improvements, health education and blood donor screening programs [7]. Much of the success of transmission-control stems from the Southern Cone [8], Central American [9] and Andean Pact Initiatives [10]. These strategies have had considerable success in interrupting vectorial transmission and have reduced new cases of the disease in many countries. However, the wide use of insecticides has created resistant triatomine populations [11]. Furthermore, large-scale vector eradication programs have suffered due to funding issues in countries such as Argentina [12]. The reduced effectiveness of insecticide-based programs in peridomestic habitats has resulted in incomplete eradication of the vector. Re-emergence of Chagas disease is an immediate threat. The need for new methods of disease control are highlighted by the emergence of drug resistant *T*. *cruzi*, toxic side-effects of available drug treatments, the inability to treat chronic disease, insecticide resistance in target vector populations and ineffectiveness of the insecticide-based approach in eliminating the disease

Paratransgenesis is an alternative approach under development to reduce transmission of vector-borne diseases by eliminating carriage of pathogens by their arthropod vector hosts [13]. The strategy focuses on understanding the microbial flora of the insect host at the developmental locus of the pathogen and genetically transforming selected bacteria to produce molecules that disrupt the target pathogen. Thus the bacteria act as a carrier or delivery agent – a Trojan horse – for the anti-pathogen molecules [14]. We developed the paratransgenic strategy to control Chagas disease transmission by triatomine bugs over 10 years ago [15]. In this strategy, the relationship between an important vector, *Rhodnius prolixus*, and its gut symbiont, *Rhodococcus rhodnii*, was exploited to disrupt the transmission of *T*. *cruzi*. *R*. *rhodnii* is a nocardiform actinomycete that aids *R*. *prolixus* with vitamin metabolism after its blood meal [16]. These actinomycetes, obtained through coprophagy, are essential for the survival of the triatomine bugs. Aposymbiotic nymphs of *R*. *prolixus* do not reach sexual maturity [17]. Because of its symbiotic association and proximity to the infective trypomastigote form of *T*. *cruzi* in the insect hindgut, *R*. *rhodnii* serves as an ideal organism for application in paratransgenesis [18]. We initially demonstrated that genetically engineered *R*. *rhodnii* that expressed an anti-trypanosomal peptide, cecropin A, when introduced into *R*. *prolixus*, resulted in elimination or significant reduction of *T*. *cruzi* in experimentally infected bugs [15]. More recent *in vitro* studies have shown that several anti-microbial peptides (AMPs) are even more effective at targeting *T*. *cruzi* when used in combination [19]. This opens up the possibility of using multiple molecules synergistically to eliminate vector carriage of the parasite.

In this study, we investigate the potential role of endoglucanases as anti-trypanosomal agents. The surface of *T*. *cruzi* is covered by a thick coat of mucin-like glycoproteins. Many of these glycoproteins are developmentally regulated and have been proposed to play a role in the binding of the cell body and the flagellum of *T*. *cruzi* to the membrane surface of the vector gut, an integral step in *T*. *cruzi* maturation [20]. We hypothesize that disruption of the glycoconjugates by endoglucanases could arrest parasite development in the vector and abort the transmission cycle. *Arthrobacter luteus* lyticase is a complex endoglucanase consisting of β-1,3-glucanase and alkaline protease that degrades β-1,3 and 1–6 glycosidic linkages [21]. We previously demonstrated that purified lyticase from *Arthrobacter* was highly effective at lysing *T*. *cruzi in vitro*. Here we report that recombinant *Arthrobacter luteus* β-1,3-glucanase expressed via *R*. *rhodnii* effectively kills *T*. *cruzi* Sylvio II strain, suggesting that this molecule may be a powerful addition to the arsenal of effector molecules for paratransgenic control of Chagas disease.

## Methods

### Sub-cloning of the β-1,3-glucanase gene into E. coli/R. rhodnii shuttle vector

The cDNA of β-1,3-glucanase from *Oerskovia xanthineolytica* (also known as *Arthrobacter luteus* strain 73–14) was a gift from Dr. Steve Slilaty [22]. To facilitate subsequent cloning steps the 1.7 kb β-1,3-glucanase cDNA was excised from pOP95-15 by AgeI and SmaI digestion, and subcloned into the XmaI and EcoRV sites of pBlueScript KS, generating pBS-glucanase.

The plasmid pRrExpA is an *E*. *coli*/*R*. *rhodnii* shuttle vector. Protein expression from this plasmid is under the control of a heterologous promoter-signal peptide complex derived from the alpha antigen gene (MKα) of *Mycobacterium kansasii*. A modified pBlueSript SK MCS, lacking the EcoRV through KpnI sites, is located just downstream of this promoter. The β-1,3-glucanase cDNA was ligated in frame into the BamHI/SmaI sites of pRrExpA following excision from pBS-glucanase by HindIII digestion, Klenow fill-in and subsequent BamHI digestion, to generate pRr-glucanase. Transformants were selected on Luria-Bertani (LB) plates containing 50 μg/ml of carbenicillin (CAR). Plasmid DNA isolated by Qiagen columns from the clones was sequenced to verify insertion.

### Transformation of pRr-glucanase into R. rhodnii

*R*. *rhodnii* electro-competent cells were generated by growing the cells in 500 mls of modified BHI (BHI with 0.1% Tween-80 and 2% glycine) at 30°C until mid-log phase. Cells were chilled on ice and harvested by centrifugation. The pellets were washed three times with a cold filtered-sterilized solution of 10% glycerol, 0.05% Tween-80, and resuspended in 1 ml of cold 10% glycerol after the final wash. These electrocompetent cells were stored as 100 μl aliquots at -80°C until ready for use.

For electroporation, competent cells were thawed on ice and mixed with 200 μg of pRr-glucanase DNA. 80 μl of this mixture were transferred to a chilled electroporation cuvette (Bio-Rad, 0.1 cm gap) and electroporated twice using a field strength of 18.5 kV/cm with a time constant of 5 ms on a BioRad Gene Pulser II. Samples were placed on ice between electrical pulses. Following electroporation, cells were allowed to recover in 1 ml of BHI at room temperature for 2 hours. 200 μl aliquots were spread onto BHI plates containing 25 μg/ml CAR. Plates were incubated at 28°C, and checked every 2 days for growth for up to 1 month.

### Selection and confirmation of R. rhodnii-glucanase clones

Plasmid specific primers, Mkα5’ (5’ CAG AGC TGA GCG GGA AGA TTC 3’) and OriR3’ (5’ GCA CGA CCA CAG CAA TAC 3’), were used for colony PCR screening of putative *R*. *rhodnii* transformants. Cycling conditions were an initial denaturation step for 3 mins at 95°C, followed by 30 cycles at 92°C for 30 sec, 54°C for 30 sec, and 72°C for 1 min, followed by final extension at 72°C for 3 min. PCR products were analyzed by electrophoresis on 1% agarose gels.

*Rhodococcus* specific 16S rDNA sequencing was performed to confirm the identity of each transformant. Genomic DNA (gDNA), isolated from each *R*. *rhodnii* transformant (MasterPure Gram Positive DNA isolation kit - Epicentre), was amplified using 16s-RrF (5’ CTG GGT CTA ATA CTG GAT ATG 3’) and 16s-RrR (5’ TGC CAT TAC TAG CGA CTC 3’) primers. Cycling conditions were 3 mins of denaturation at 95°C, followed by 30 cycles of 95°C for 30 sec, 52°C for 30 sec and 72°C for 1.5 min, followed by a final extension for 3 mins at 72°C. Amplification was confirmed by running a portion of the reaction mixture on a 1% agarose gel. Samples that amplified were subsequently purified from the reaction using the Qiagen PCR Purification kit per manufacturer’s instructions, and sequenced using the PCR primers described above with the BigDye Terminator Reaction Cycle Sequencing Kit (Applied Biosystems). Sequences were then submitted for BLAST analyses at the GenBank database.

### Production of cell extracts and spent media from R. rhodnii transformants

Cells from 100 ml mid-log cultures of transformed and untransformed (negative control) *R*. *rhodnii* were pelleted by centrifugation and the spent media frozen at -20°C. The pelleted cells were suspended in 10 ml of complex lysis buffer (100 mM Tris, 500 mM NaCl, 0.5 mM EDTA, 0.1% Triton X-100, 0.1% Tween-20, 8% glycerol, 250 mM urea, 5 mM 2-mercaptoethanol, 100 μg/ml PMSF, 1X HALT Protease Inhibitor Cocktail, 50 μg/ml lysozyme and 1U benzonase) and lysed by alternating the suspended cells between cooling on ice and vortexing at ten-minute intervals. Following complete lysis after 30 mins, cellular debris was removed by centrifugation at 15,000 rpm for 30 mins. Half of the lysate was flash-frozen and stored at -80°C. The remainder was dialyzed for 48 hours at 4°C into 1X TBS (25 mM Tris, 0.5 M NaCl, pH = 7.2) with Sigma protease inhibitors using a 2,000 MWCO Slide-A-Lyser dialysis cassette (Pierce), with one change of dialysis buffer at 24 hours. Dialysate was aliquoted, flash-frozen and stored at -80°C.

### Characterization of polyclonal antibody to β-glucanase

A rabbit polyclonal antibody to the CHEPGTQFRGRVDGD epitope of β-glucanase was produced by GenScript for this laboratory. The specificity and affinity of this affinity-purified antibody was determined via ELISA against *Arthrobacter* lyticase complex and the synthetic peptide used to produce the primary antibody. Five-log dilutions of lyticase (100 U/μl to 0.01 U/μl) or β-glucanase peptide (0.01 nM to 100 nM) were bound overnight at 4°C to 96-well plates. Wells were washed and blocked with 2.5% BSA in TBS-T (25 mM Tris, 0.5 M NaCl, pH = 7.2, 0.5% Tween-20), and the primary antibody was added at a 1:5000 dilution in TBS-T. After a 2 hour incubation at 37°C, the primary antibody was removed, wells washed as before and a 1:5000 dilution of anti-rabbit IgG HRP added. Plates were incubated at 37°C in the dark for one hour before being washed 3 times prior to detection with single component TMB (BioRad). Plates were then read at OD_450_ on a Molecular Devices SpectraMax M2 Fluorescent microplate reader. These experiments were repeated in triplicate.

### Western analysis of cell lysates and spent media

Lysates and spent media from transformed and control *R*. *rhodnii* were separated using SDS-PAGE on 8% Tris-glycine gels (Invitrogen). Proteins were subsequently transferred to 0.2 μm pure nitrocellulose membranes. Subsequent to blocking with 5% non-fat dried milk in TBS-T, a 1:5000 dilution of the anti-glucanase antibody was added and the membrane incubated overnight at 4°C. Following three washes, a secondary anti-rabbit IgG coupled to HRP was added to the membrane at a 1:10,000 dilution for 1 hour at room temperature. Blots were developed by incubation in TMB.

### ELISA of cell extracts and spent media

Samples from transformed and control *R*. *rhodnii* diluted in TBS-T were added to triplicate wells on microtiter plates, and incubated for 2 hours at 37°C. After washing the wells in TBS-T, 2.5% BSA was added to block for 1 hour at 37°C. The primary antibody (GenScript USA), diluted to 1:5000 (in TBS-T), was removed after overnight incubation at 4°C. The secondary antibody used in these experiments was an anti-rabbit IgG coupled to HRP. 100 μl of a 1:5000 dilution of this antibody was added to each well, and allowed to incubate at 37°C for an hour in the dark. Detection of expressed recombinant protein was accomplished after 3 final washes by development in TMB. Plates were read at 450 nm on the Molecular Devices SpectraMax M2 Fluorescent Microplate Reader.

### Toxicity of recombinant β glucanase against T. cruzi

The toxicity of recombinant β glucanase produced by *R*. *rhodnii* in cell lysates and spent media against epimastigote cultures of *T*. *cruzi* Silvio II (ATCC #50823) was determined by a combination of spectrophotometric turbidity and live/dead fluorescent microscopy assays as described in Fieck *et al*. [19]. Briefly, lysates from β glucanase recombinants were diluted in 1X PBS and added to triplicate wells of sterile tissue culture certified 96-well plates. 10^5^ parasites were added to each well and the plates incubated at 28°C for a total of 96 hours. The optical density of the cultures was measured at 600 nm every 24 hours. After the incubation period, parasites from triplicate wells were pooled and stained for 1 hour with 1 μl of 5 mM Calcein-AM (Molecular Probes). After washing in 1.0 ml 1X PBS, brightfield and green fluorescent images were generated on a Nikon Eclipse 80i microscope. In these experiments, lysates and spent media from untransformed *R*. *rhodnii* were used as negative controls. No human subjects or animals were used in this study.

## Results

### Construction of pRr-glucanase and transformation of R. rhodnii

Sequence analysis revealed that the β-1,3-glucanase cDNA was successfully cloned downstream of the MKα promoter-signal peptide of the pRrExpA shuttle vector. This clone, pRr-glucanase, was subsequently electroporated into *R*. *rhodnii*. The transformation efficiency of actinomycetes was in the range of one transformant per 10^8^ cells. A total of 40 putative *R*. *rhodnii*-glucanase transformants were selected after 3 days of growth on BHI plates containing 25 μg/ml CAR. Of these, four were randomly selected for further characterization. The remaining cells were propagated in BHI containing 25 μg/ml CAR and cryopreserved in 10% glycerol for future use.

Amplification of the 16S rDNA using *Rhodococcus* specific primers on isolated genomic DNA confirmed that the selected transformants were, indeed, *R*. *rhodnii*. A 1.4kb PCR product was obtained from two of the four selected clones. Sequencing of the PCR product confirmed that the recombinant organism was *R*. *rhodnii*. To confirm that the two remaining *Rhodococcus* transformants harbored pRr-glucanase, colony PCR was performed using plasmid specific primers that flank the insert. Only one of the four transformants yielded a 1.7 kb fragment that is indicative of the insert.

### Characterization of anti-β-1,3-endogluconase polyclonal antibody

**Figure 1:**
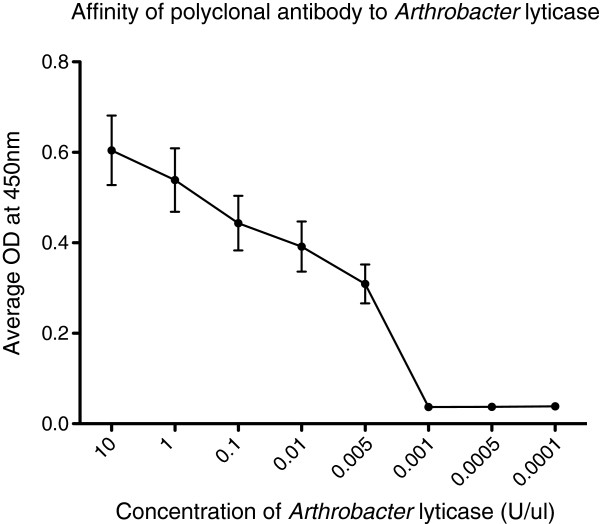
**Binding affinity of polyclonal anti-glucanase antibody. **The binding affinity of the polyclonal anti-glucanase antibody at a dilution of 1:5000 was determined against five-log dilutions of *Arthrobacter *lyticase. Note that this polyclonal antibody can detect β-glucanase in as little as 0.05 U/μl of *Arthrobacter *lyticase. Each point represents the mean with standard error bars of triplicate samples. Experiments were performed at least twice.

**Figure 2:**
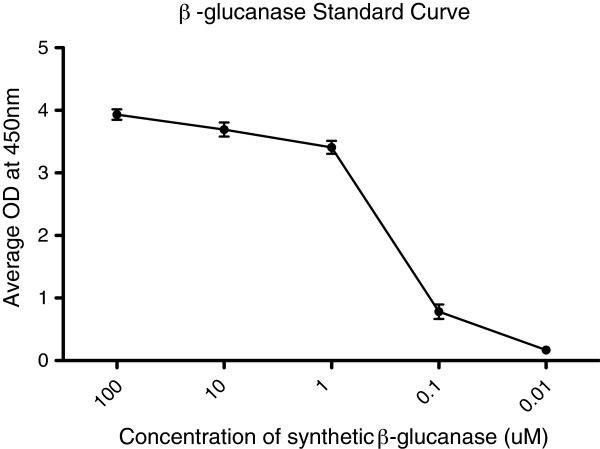
**ELISA standard curve for β-glucanase.** Decreasing concentrations of synthetic β-glucanase were titrated against 1:5000 dilution of the polyclonal anti-glucanase antibody to generate ELISA standard curves such as the one shown here. These curves were used to quantify recombinant β-glucanase expression from each *R*. *rhodnii* clone. All points represent the mean with standard error bars of triplicate samples. Experiments were performed at least twice.

After a thorough and unsuccessful search for commercially available antibodies against β-glucanase, a synthetic antibody was specifically raised for the detection of this recombinant molecule. The affinity of this antibody was initially tested by examining its ability to bind commercially available *Arthrobacter* lyticase. Figure 1 shows that a 1:5000 dilution of this antibody is capable of detecting *Arthrobacter* lyticase at a limit of 0.005 units/μl. The antibody was synthesized against an epitope on the β-glucanase peptide that is part of the *Arthrobacter* lyticase complex. Therefore, synthetic β-glucanase peptide was used to generate a standard curve that could be used to determine the expression level of glucanase from transformed *R*. *rhodnii* (Figure 2). The antibody detected β-glucanase at concentrations as low as 10nM. Resolution of concentrations lower than 10nM was impossible from the present ELISA. The OD readings corresponding to these standards curves were used to calculate the concentration of recombinant β-glucanase from the *R*. *rhodnii* clones.

### ELISA analysis of lysates and spent media

**Figure 3:**
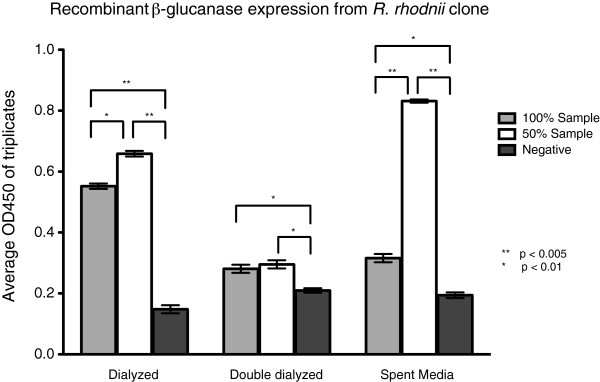
**Expression of β-glucanase from *R. rhodnii *Clone 1. **Dialyzed lysates and spent media from transformed *R. rhodnii* was examined for expression of β-glucanase. Expression of β-glucanase was consistently higher in each of the diluted samples, suggesting that there is self-blocking at high protein expression levels. As expected, the expressed protein is found predominantly in the spent media. Bars represents the mean with standard error bars of triplicate samples wells.

After standardization, lysates and spent media from cultures of the *R*. *rhodnii* transformant were analyzed via ELISA to confirm expression of recombinant β-glucanase. The concentrations of recombinant β-glucanase were extrapolated from the standard curve of the synthetic peptide. ELISA detected β-glucanase at a lower threshold of approximately 100 nM from 0.5X spent media and 80 nM from cellular lysates (Figure 3). Interestingly, 0.5X dilution of the spent media and lysates revealed a higher concentration of recombinant peptide than undiluted samples (Figure 3). We conducted another set of experiments to explain this finding. (1) To remove the effect of the lysis buffer, samples were dialyzed twice. Double dialysis led to loss of the recombinant protein (Figure 3) and was not a viable method of purification; (2) ELISA revealed that non-specific binding of the dilution buffer by the antibody was not a factor (data not shown); (3) ELISA using synthetic β-glucanase peptide added to wild-type *R*. *rhodnii* cell extracts showed that cell extracts block the signal from synthetic β-glucanase peptide (data not shown). These experiments suggest that the higher signal observed in diluted samples of the cell lysate is due, in part, to dilution of the cellular extract, which enabled detection of the recombinant peptide. Since the spent media samples were not affected by cellular extracts, the increase in signal in these samples after dilution was due to the prozone or hook effect observed in many immuno-ligand binding assays [23].

### Toxicity assays

**Figure 4:**
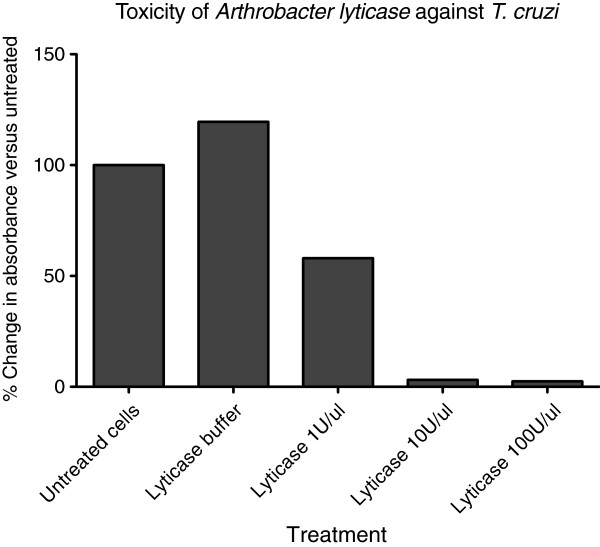
***Arthrobacter *lyticase inhibited the growth of *T. cruzi *Sylvio. ***T. cruzi *Sylvio was cultured in media with indicated concentrations of *Arthrobacter *lyticase. Parasite turbidity was determined following 96-hour incubation and compared to untreated parasites. Results were averaged from triplicate samples in two independent experiments, and are presented as the percent change in turbidity between treated and untreated cultures following the 96-hour incubation.

**Figure 5:**
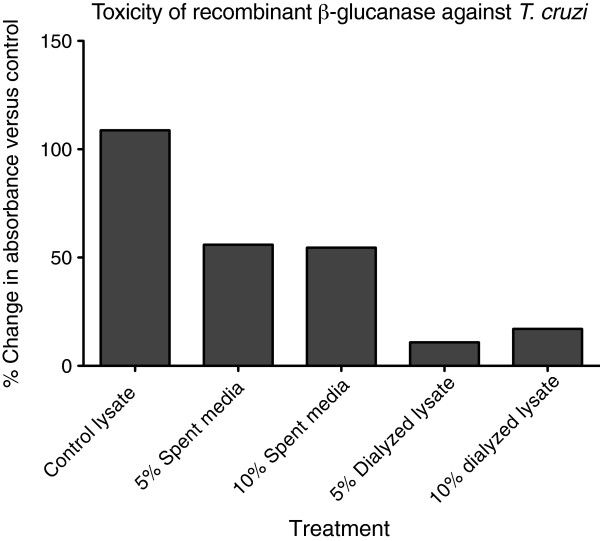
***T. cruzi *Sylvio is inhibited by recombinant β-glucanase. **Indicated amounts of dialyzed lysates or spent media, containing recombinant β-glucanase, harvested from transformed *R. rhodnii *were added to cultures of *T. cruzi *Sylvio. Turbidity of parasites treated with lysates from untransformed *R. rhodnii* (control lysate) was compared to that of the treatment group after 96-hours. Results are presented as the percent change in turbidity between treated and untreated cultures following this incubation period. The result shown here is a representative from two independent experiments.

*Arthrobacter* lyticase exerted approximately 50 percent growth inhibition of *T*. *cruzi* at the lowest concentration applied (1 U/μl) and complete inhibition at 10 and 100 U/μl (Figure 4), demonstrating that lyticase is lethal to *T*. *cruzi* at very low concentrations. Recombinant peptide samples were therefore diluted to 5% and 10% for toxicity studies. As seen in Figure 5, recombinant β-glucanase isolated from filtered spent media inhibited *T*. *cruzi* growth by 50% at both dilutions. Filtered lysates containing recombinant β-glucanase were also diluted to 5% and 10%. These samples inhibited *T*. *cruzi* growth by ~80% without any significant difference in the growth inhibition between the two dilutions (Figure 5). Lyticase buffer and lysates from untransformed *R*. *rhodnii* (negative controls) showed 0% inhibition of *T*. *cruzi* growth (Figures 4 and 5).

**Figure 6:**
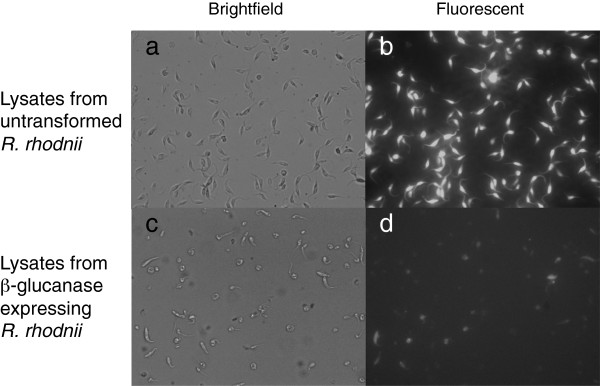
**Microscopic analysis of *T. cruzi *Sylvio following treatment with recombinant β-glucanase.***T. cruzi *Sylvio were treated with either cell lysates from untransformed *R. rhodnii*, or dialyzed lysates containing recombinant β-glucanase. Following 96-hour treatment, the parasites were harvested, stained with Calcein-AM and examined at 40X.

Microscopy studies confirmed the results we observed from spectrometric toxicity assays. Brightfield and fluorescence microscopy demonstrated a significant reduction in the number of live *T*. *cruzi* cells after they were treated with recombinant β-glucanase. Figure 6 shows the microscopy images of *T*. *cruzi* treated with lysates isolated from untransformed *R*. *rhodnii* alongside *T*. *cruzi* treated with lysates from β-glucanase-expressing *R*. *rhodnii*. Bright field images show a marked decrease in number of *T*. *cruzi* after treatment with recombinant β-glucanase. Most of the cellular debris seen under the bright field after treatment do not fluoresce nor have the structural integrity of healthy *T*. *cruzi* suggesting that they are lysed, unviable remnants of *T*. *cruzi*. Additionally, since only live cells take up the fluorescent Calcein AM and we see a decrease in fluorescent bodies after treatment, we can conclude that the treatment is effective in lysing *T*. *cruzi*.

## Discussion and conclusions

β-glucanase is a stable molecule with cruzicidal activity and, therefore, may play a part in a paratransgenic strategy for the control of Chagas disease. Given the critical role of the glycocalyx of *T*. *cruzi* in parasite development within the arthropod gut, we believe that disruption of the sugar coat using this molecule will abort vectorial transmission. Here we report the development of transformed *R*. *rhodnii* that express recombinant β-glucanase. This was confirmed by the results from ELISA of lysates and spent media isolated from an engineered line of this symbiont. Our results suggest that the cellular lysates contain a higher concentration of recombinant β-glucanase than surrounding media. We observed that the blocking effect of the cellular extract combined with a marked prozone phenomenon resulted in under-estimation of the concentration of recombinant β-glucanase in cell lysates. Nevertheless, toxicity assays revealed that any effect of *T*. *cruzi* inhibition can be attributed entirely to the recombinant β-glucanase. In ongoing studies, we are developing expression systems for *R*. *rhodnii* that permit enhanced secretion of heterologous proteins. We expect these constructs to improve the efficacy of β-glucanase as an effector molecule in the gut of triatomine bugs. However, high rates of bacterial turnover in the gut of these bugs, especially after blood meals, will liberate recombinant molecules as well [15,24].

Development of recombinant β-glucanase as an effector for paratransgenesis adds another molecule to our growing arsenal. *In vitro* studies have already established that AMPs used in combination have synergistic lethal effects against *T*. *cruzi*[19]. β-glucanase could be a powerful addition to the system when used in combination with other molecules. Drug resistance of *T*. *cruzi* has been a major problem with treating Chagas disease and similar resistance could evolve to molecules that are delivered by paratransgenic mechanisms. Use of multiple effector molecules would help reduce development of resistance. AMPs target cellular membranes and internal components to exert toxicity while β-glucanase works on sugar moieties sequestered on the surface of the parasite. Dual targeting of *T*. *cruzi* should not only slow resistance but also permit synergistic or additive effects on *T*. *cruzi* lethality. We have also developed recombinant single chain antibodies that further strengthen the tool chest [25]. These single chain antibodies, which target sialic acid epitopes on *T*. *cruzi* surfaces, will be used in an integrated and comprehensive field-based strategy to target vector stages of *T*. *cruzi*, as part of paratransgenic control of Chagas disease.

Currently, we are conducting *in vivo* co-infection studies in *R*. *prolixus* nymphs using engineered lines of *R*. *rhodnii* to study the effects of our molecules on the arthropods and verify efficacy. Aposymbiotic nymphs are infected with transformed *R*. *rhodnii*, allowed to develop, and challenged with *T*. *cruzi* in a blood meal. We have set up *in vivo* studies that examine the effects of AMP combinations and recombinant β-glucanase on arthropod maturation, fecundity and competence to carry and permit maturation of *T*. *cruzi*. Successful colonization of *R*. *prolixus* nymphs by the transgenic symbionts and elimination of *T*. *cruzi* carriage in all paratransgenic insects will set the stage for simulated field trials.

Chagas disease remains a public health concern and an important neglected disease. Despite efforts by governments and overall improvement in living conditions, detection, and treatments, tens of millions of people are exposed to the disease-causing parasites annually. Current methods involving insecticides as a method of vector control work only in small domestic areas and carry high costs. The paratransgenic method using recombinant symbiotic bacteria may serve as a valuable adjunct in the ongoing battle to eliminate Chagas disease.

## Abbreviations

BHI: Brain heart infusion medium; BSA: Bovine serum albumin; CAR: Carbenicillin; ELISA: Enzyme-linked immunosorbent assay; gDNA: Genomic DNA; LB: Luria-bertani medium; LIT: Liver infusion tryptose medium; MKα: *Mycobacterium kansasii *alpha antigen; MWCO: Molecular weight cut-off; PBS: Phosphate buffered saline; PCR: Polymerase chain reaction; TBST: Tris-buffered saline with 0.5% tween-20.

## Competing interests

The authors declare that they have no competing interests.

## Authors’ contributions

CJ, NK, SW, AF and IH contributed to all the experiments described in this paper. CJ wrote the manuscript. AF, IH and RD analyzed and interpreted the results. IH and RD finalized the manuscript in consultation with the other authors. All authors revised and approved the final version of this manuscript.
